# Ending Neonatal Deaths From Hypothermia in Sub-Saharan Africa: Call for Essential Technologies Tailored to the Context

**DOI:** 10.3389/fpubh.2022.851739

**Published:** 2022-04-07

**Authors:** Giorgia Brambilla Pisoni, Christine Gaulis, Silvan Suter, Michel A. Rochat, Solomzi Makohliso, Matthias Roth-Kleiner, Michiko Kyokan, Riccardo E. Pfister, Klaus Schönenberger

**Affiliations:** ^1^École Polytechnique Fédérale de Lausanne, EssentialTech Centre, Lausanne, Switzerland; ^2^Clinic of Neonatology, Department Women-Mother-Child, Lausanne University Hospital, University of Lausanne, Lausanne, Switzerland; ^3^Neonatal and Paediatric Intensive Care Unit, University Hospitals of Geneva and Geneva University, Geneva, Switzerland

**Keywords:** Sub-Saharan Africa (SSA), newborn, mortality rate, hypothermia, incubators, low weight at birth, prematurity

## Abstract

Neonatal death represents a major burden in Sub-Saharan Africa (SSA), where the main conditions triggering mortality, such as prematurity, labor complications, infections, and respiratory distress syndrome, are frequently worsened by hypothermia, which dramatically scales up the risk of death. In SSA, the lack of awareness on the procedures to prevent hypothermia and the shortage of essential infant devices to treat it are hampering the reduction of neonatal deaths associated to hypothermia. Here, we offer a snapshot on the current available medical solutions to prevent and treat hypothermia in SSA, with a focus on Kenya. We aim to provide a picture that underlines the essential need for infant incubators in SSA. Specifically, given the inappropriateness of the incubators currently on the market, we point out the need for reinterpretation of research in the field, calling for technology-based solutions tailored to the SSA context, the need, and the end-user.

## Introduction

Among the 17 Sustainable Development Goals (SDGs) conceived in 2015 by the 2030 Agenda for Sustainable Development^1^, SDG3 deals with ensuring health and promoting well-being for all, at all ages. In particular, SDG3.2 aims at reducing the mortality rate of children below 5 years of age (i.e., under-five; 25 deaths per 1,000 live births by 2030), including newborns (i.e., 1–28 days of age; 12 deaths per 1,000 live births by 2030)^2^, An overall reduction of the under-five mortality has been achieved worldwide, especially in high-income countries (HICs), however, low- and middle-income countries (LIMCs) are still far in the process of meeting the mortality rate defined by SDG3.2 (1). Of note, in LMIC settings, the COVID-19 outbreak has strongly compromised the health scenario, therefore, a substantial rise in child deaths is expected in the upcoming years (2).

Sub-Saharan Africa (SSA)^3^ is the region worldwide displaying the highest under-five and neonatal mortality rates (76 and 27 deaths per 1,000 live births in 2019, respectively) (3). SSA accounts for almost half (~42%) of the global neonatal deaths occurring every year (3); in fact, of the global 2.45 million newborn deaths of 2019, roughly 1 million have died in SSA solely (4).

Prematurity (~35%), asphyxia (~24%), infections (~23%), and congenital anomalies (~11%) are considered the leading causes of neonatal mortality (5). In developing countries, and particularly in SSA, neonatal hypothermia strongly increases the risk of death (6). Neonatal hypothermia, defined by the WHO as the neonatal thermal state by which the body temperature falls below 36.5°C (7), is conventionally, classified into three main sub-groups: cold stress (36.4–36.0°C), moderate hypothermia (35.9–32.0°C), and severe hypothermia (<32.0°C) (7).

Despite almost never considered the cause of death *per se*, hypothermia highly increases the risk of neonatal mortality when concomitant with one of the aforementioned leading causes of death (6, 8). In SSA, newborns are at particularly high risk of hypothermia due to a mix of physiological features (i.e., high incidence of low-weight at birth and prematurity), cultural beliefs (i.e., delayed breastfeeding/skin-to-skin contact/drying and wrapping, late hospital admission, newborn bathing/oil massage right after birth), and socioeconomic factors (i.e., high prevalence of home deliveries, absence of skilled care at delivery and unavailability of medical devices, including for post-delivery transport, poverty, and out-of-pocket payments for health services). These factors merge with a critical health care condition of shortage in essential infrastructures (i.e., neonatal units and equipment) and a lack of awareness of the risks associated to hypothermia (i.e., absence of skilled personnel and insufficient or absent training for hypothermia prevention and treatment) (9–13).

Moreover, in the resource-depleted settings of SSA, neonatal death registries are often incomplete and inaccurate, live births under-reported, causes of death misclassified, and newborn body temperature often unmeasured (6, 8, 14). It comes with no surprise, hence, that the epidemiological prevalence of neonatal hypothermia is difficult to evaluate in these countries, with estimates ranging from 32 to 85% in hospital-born infants, and from 11 to 92% for home deliveries (6). Alongside, the prevalence of hypothermia in SSA hospitals spans from 8% within the first 12 h after birth to 85% in the case of hospital admission (13). In general, such broad intervals reflect a lack in the knowledge about the incidence and prevalence of hypothermia, both from a clinical and an epidemiological perspective (6). However, this gap in the knowledge should not lead to underestimate the burden of hypothermia, which is indeed a “silent epidemic” (10).

The first set of thermal care guidelines to prevent and treat hypothermia has been assembled by WHO in 1997 and referred to as the warm chain. The warm chain consists of 10 essential measures, which are: (i) warm delivery room, (ii) prompt newborn drying, (iii) skin-to-skin contact or Kangaroo Mother Care (KMC), (iv) prompt breastfeeding, (v) postponement of bath and weighing, (vi) appropriate clothing and bedding, (vii) promotion of mother-baby contact, (viii) warm transportation, (ix) warm resuscitation, and (x) awareness and adequate training (7). Despite these guidelines, neonatal hypothermia remains a major determinant of infant death in SSA countries. Indeed, some measures may be insufficient or unpractical in the SSA context (9–13).

In this article, we present a description of the current practices and tools to prevent and treat hypothermia, focusing on the medical devices available on the market. We also include a snapshot on conceptual and technological development of infant incubators throughout history. We focus on the SSA context for a comprehensive summary of thermal practices and equipment for neonates, with a particular focus on Kenya. Our aim is to uncover bottlenecks in technology-based thermal solutions to help reduce neonatal mortality. We propose a model to reinterpret the current body of evidence and foster a context- and end user-informed research in the field of essential technologies for newborns.

### Search Strategy and Selection Criteria

We searched PubMed and MEDLINE for peer-reviewed articles published in English between 1997 [WHO definition of neonatal hypothermia, (7)] and 2021. An initial search (papers identified are {in brackets}) has been conducted using the following keywords: “newborn AND hypothermia” {3553}, “neonatal mortality AND hypothermia” {1030}, “neonatal hypothermia AND prevalence” {776}. Afterwards, inclusion criteria about the relevant context (i.e., “newborn AND hypothermia AND developing countries” {85}, “neonatal mortality AND hypothermia AND developing countries” {52}, “neonatal hypothermia AND prevalence AND developing countries” {39}, “neonatal hypothermia AND Sub-Saharan Africa” {109}) and the year of publication (1997–2021 range selected) have been included. Of these 285 papers identified, the relevant ones have been cited accordingly. Of note, we have not filtered the search for “infant incubators AND hypothermia” {91} and “infant warmers AND hypothermia” {82} for time or context. International reports from humanitarian organizations (i.e., UN, UNICEF, and WHO) have been included. Book chapters, demographic data, market news, and press articles on released technologies have also been thoroughly searched on the web and included as footnotes.

Patient and Public Involvement: no patient involved.

### Description of the Nature of the Evidence Being Addressed and Rationale for the Proposed Hypothesis and Theory

#### Newborn Hypothermia: Prevention and Treatment

Clinical neonatal thermal care first relies on prevention of established hypothermia. Hypothermia prevention mostly appeals to the maintenance of an appropriate environmental temperature. In the WHO warm chain document of 1997 (7), the first recommendation focuses on warm delivery rooms. At birth, newborns are exposed to an environment at least 10°C colder than the in*-*utero (~37.5°C) maternal body temperature. As such, the WHO recommends a room temperature at delivery of 26-28°C, in most contexts warmer than the indoor room temperature range (18–24°C) (15). The second preventive recommendation in the warm chain addresses prompt drying and warm wrapping of the newborn to primarily reduce evaporative heat losses, but also convection, conduction, and radiation (15). A systematic review has for instance shown that the use of plastic bags to wrap the newborn right after birth is associated to less hypothermia and higher temperature at admission, specifically in the case of low-birth-weight newborns (16). Of note however, only 4 of the 43 studies analyzed in this systematic review concerned LMIC settings (16).

The third recommendation focuses on the close warming contact between the mother and the infant (i.e., skin-to-skin contact or KMC, alongside breastfeeding). KMC was proposed at first in 1978. It rapidly became an essential component within the standard newborn care program for infant thermal care and effective prevention of hypothermia (17). KMC consists in the uninterrupted skin-to-skin contact between the newborn and the mother's or caregiver's chest. Importantly, KMC application contributes at least to a 40% reduction in the neonatal mortality rate (18). KMC is especially recommended for clinically stable low-birth-weight and premature newborns (19–21). Of note, KMC has been shown to reduce the mortality of low-birth-weight newborns by 25% when initiated immediately after birth (22).

Despite this, however, the implementation of KMC remains slow in LMICs, mostly because of inadequate and poorly developed neonatal care units (i.e., lack of KMC-dedicated spaces, shortage of healthcare workers, beds and chairs, lack of privacy, cultural aspects, and overcrowding) (19–21).

When preventive measures are not applied or sufficient, clinical neonatal thermal care relies on treatment of hypothermia. For that, all WHO recommendations depend on the availability and good operation of technical devices for re-warming, such as incubators and radiant warmers (7).

### Essential Technologies to Treat Hypothermia: Incubators and Warmers

#### Historical Development of Infant Incubators and Warmers

**Figure 1:**
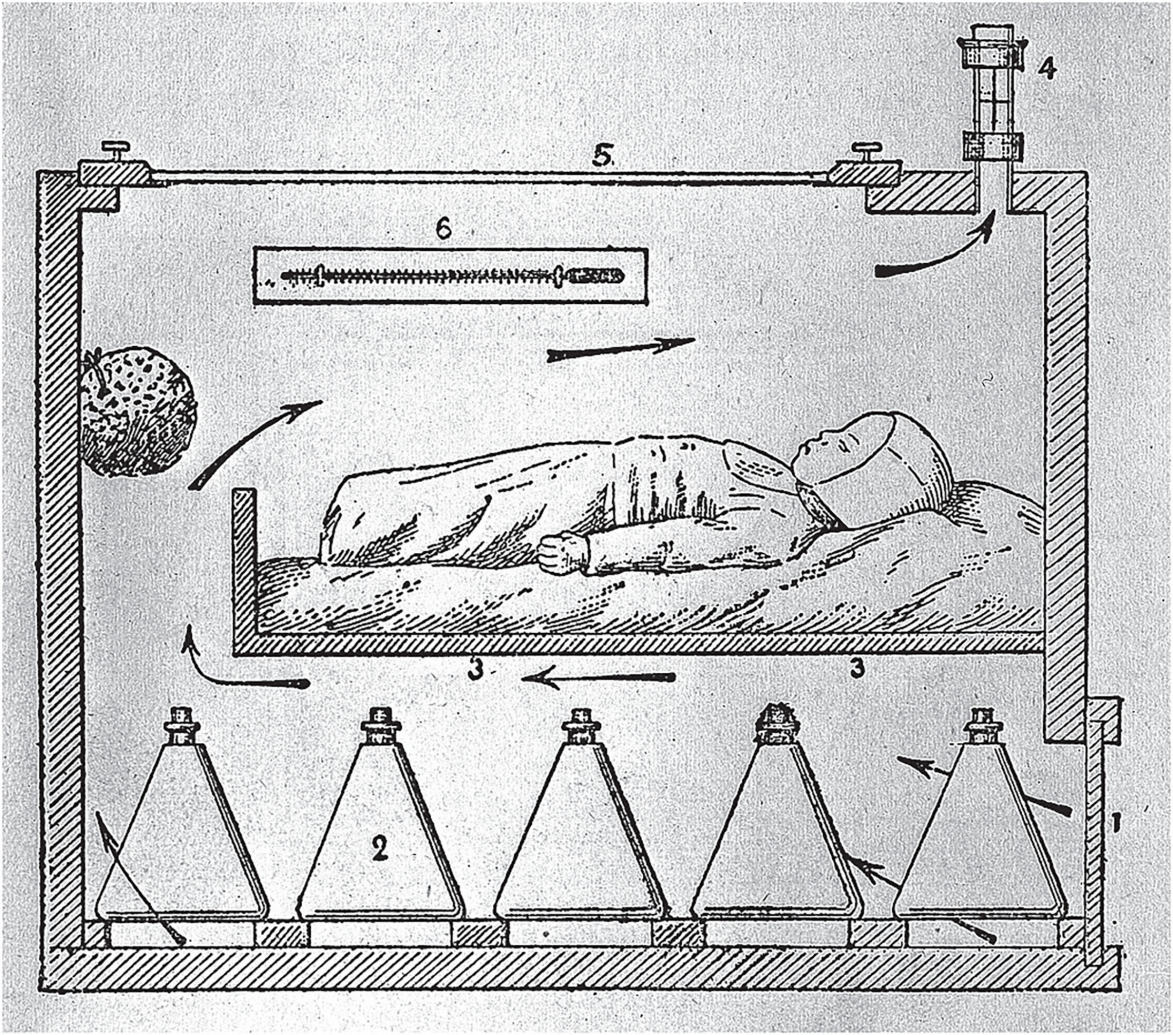
Section of Tarnier's Incubator. Retrieved from: https://commons.wikimedia.org/wiki/File:Section_of_Tarnier%27s_incubator;_Budin,_The_Nursling,_1907_Wellcome_L0005632.jpg. Arrows indicate the air flow.

The first proof of existence of an incubator for premature newborns goes back to 1722, when the Italian physician Giuseppe Liceti put together a very rudimental device inspired by the system Egyptian farmers used to assist chicken eggs to hatch (23, 24). Almost a century passed before a double-walled metal incubator was reported in Russia in 1835 by the physician Johann Georg von Ruehl (25). Despite these two earlier reports, the French physician Jean-Louis-Paul Denucé is considered the author of the first infant incubator with his design devised in 1857. Like the von Ruehl's design, Denucé's incubator was made of a double-walled metal tub heated with water. A few years later, in 1860, the German obstetrician Carl Credé and the French obstetrician Stéphane Tarnier developed similar models almost concomitantly. Credé's incubator also consisted of a double-walled metal tub, but it was warmed with water circulating externally to the chamber (26). Tarnier's incubator was more inspired by the Liceti's original design and made from a chicken incubator converted into a newborn care device (Figure 1).

**Figure 2:**
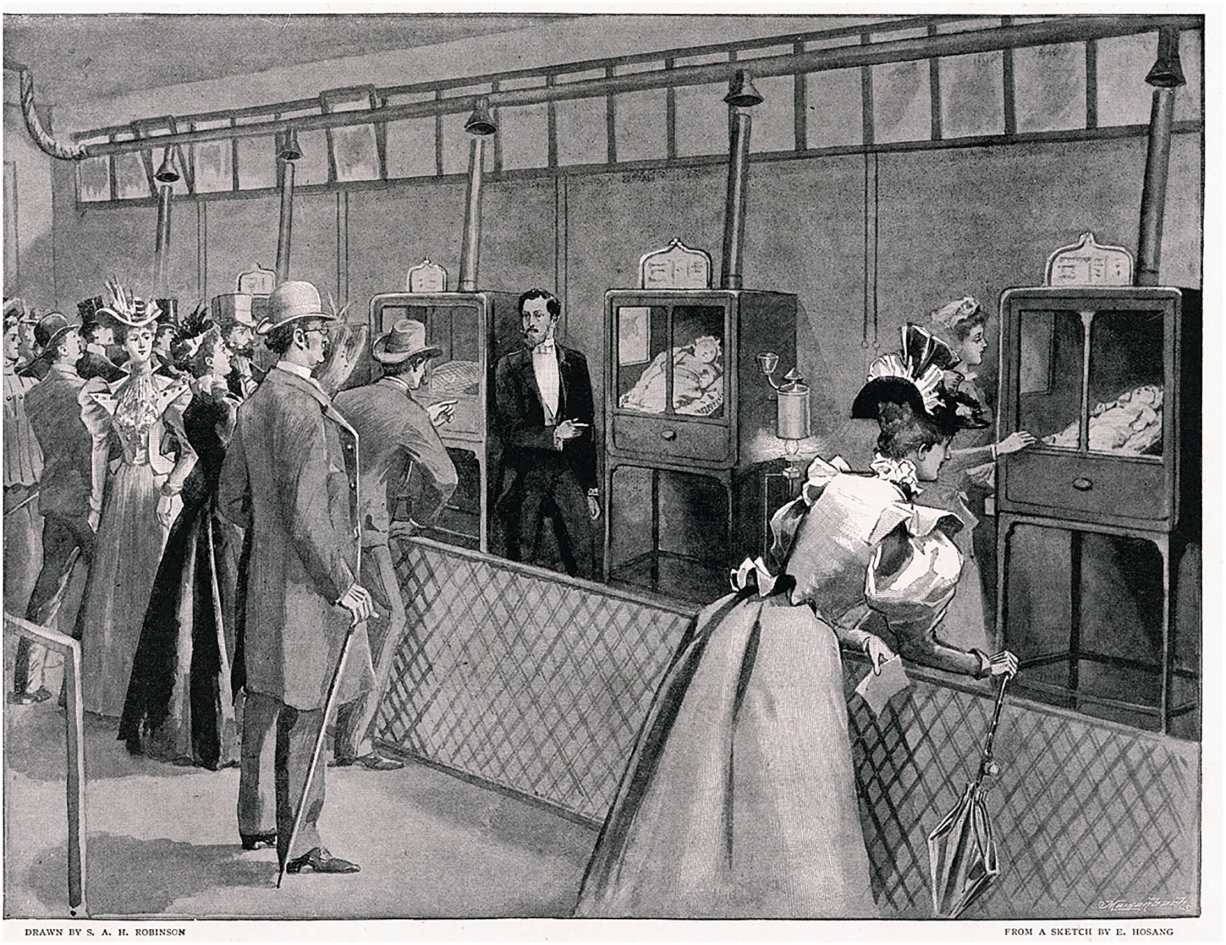
“An Artificial Foster Mother: Baby Incubators at the Berlin Exposition”. Display of Lion Incubators in 1896. Retrieved from: The Graphic 1896; 54:461; https://www.epoch-magazine.com/post/mothers-and-machines-on-the-midway-the-curious-case-of-baby-incubators.

**Figure 3:**
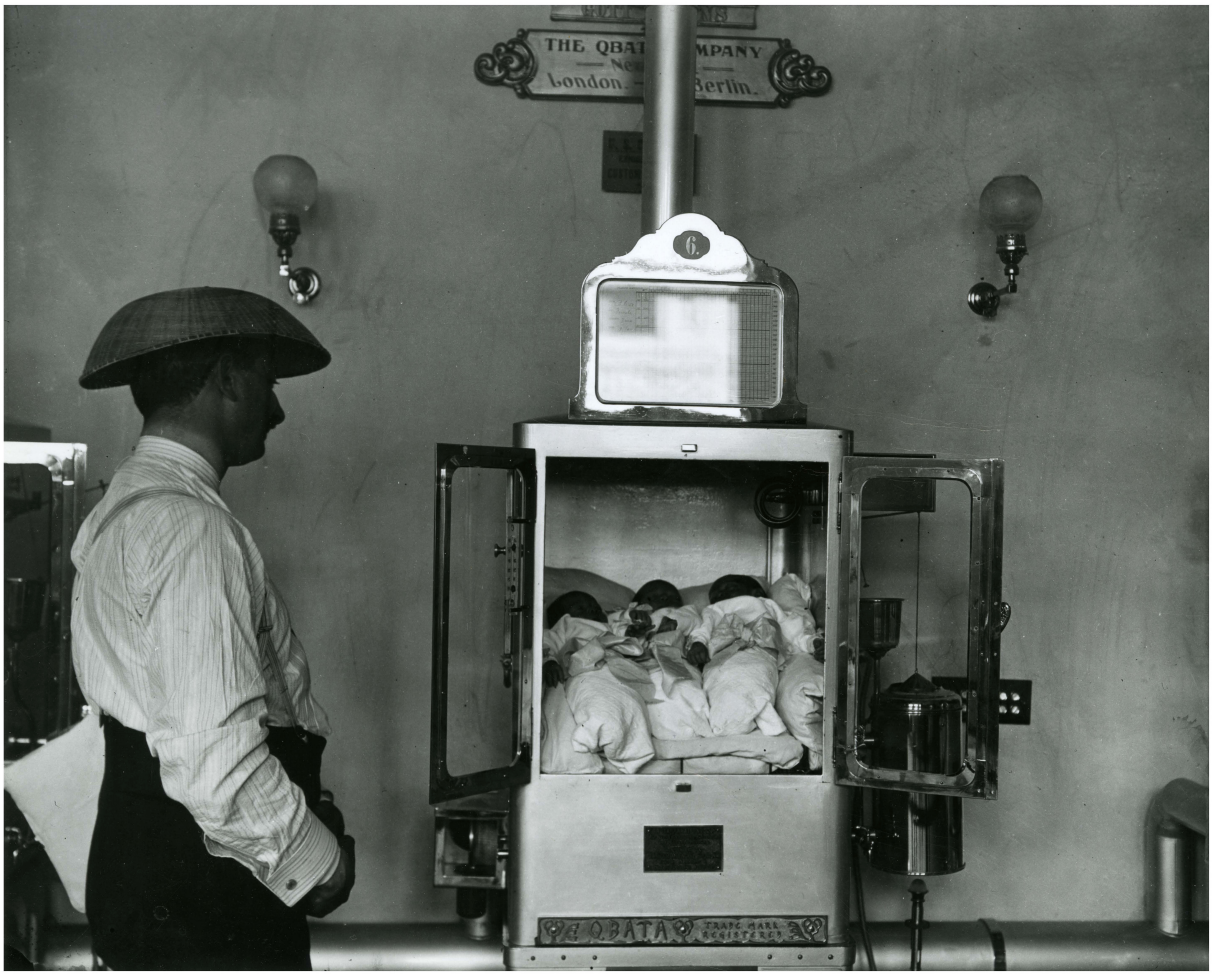
History of Medicine: The Incubator Babies of Coney Island. Retrieved from: https://columbiasurgery.org/news/2015/08/06/history-medicine-incubator-babies-coney-island.

With the beginning of 1890, a former French student of Tarnier, Pierre-Constant Budin together with his American pupil Martin Couney marked the beginning of a new era, characterized by the commercial launch of neonatal incubators and their use in exhibitions worldwide (Figure 2) (27). The incubator of Budin and Couney was the first to use air heating and even a rudimentary monitor for humidity and temperature. In 1914, Couney settled in Chicago and made the acquaintance of Dr. Julius Hays Hess, who is considered the father of American neonatology. In 1922, Hess designed, conceived, and patented different incubator prototypes (28). His first device consisted in an electrically heated bed with a water jacket and a metal hood for insulation. In 1932, Hess designed and patented a more modern design with a closed chamber equipped with an oxygen dispenser (Figure 3) (28, 29).

On a more experimental note, in 1957, the American neonatologist William Silverman started a series of large-scale randomized control trials assessing the causality between thermal care and newborn survival. These studies may well be the first randomized controlled trials in newborn care. Silverman's observations triggered the design of the first closed incubator including surveillance and servocontrol of the temperature (30, 31). High survival rates were independently reported for the Silverman incubator by three different studies performed in the following years (31). However, the subsequent commercialization of the device on a large scale led to the identification of an operational issue in terms of heat loss, such that the heating system was dismissed and substituted by a convection mechanism (32). These first incubators available for clinical use, were tested by the American neonatologist Paul Perlstein who showed unstable temperatures within servo-controlled incubators (33). By teaming up with the electrical engineer Neil Edwards and the computer programmer Harry Atherton, Perlstein worked on stabilization of incubator temperatures. In 1975, a new device called Alcyon came on the market and was submitted to a 1 year-long trial with over 200 infants (34, 35). Despite its stability in terms of temperature, the Alcyon never reached commercialization because of its complex operation and expensive design. Between 1970 and 1980, with the development of neonatal care practice (i.e., mechanical ventilation), ease of access became fundamental (36). Open incubators were conceived as open-bed devices surrounded by walls more for security reasons and warmed through radiant heat source, hence referred to as radiant warmers. However, because of humidity dispersion, these devices were not adapted for very premature neonates with immature skin and high trans-epithelial water losses (29, 37).

#### Modern Infant Incubators and Warmers: A Snapshot on the Global Market

Basically, three incubator types are on the market: open (or radiant warmers), closed and transport incubators.

Open incubators are lower priced than closed ones, but more prone to humidity dispersion. Whilst allowing easier access, open incubators do not offer a protective “bubble” around the infant. Moreover, they have high energy consumption requiring a constant and efficient electric energy supply (38).

Closed incubators are designed to provide a stable microclimate, offering an environment where temperature, humidity, and sometimes oxygen can be thoroughly regulated. In addition, the protected space guarantees some isolation from pathogens (through air filters) and noise of the surrounding environment, while still allowing access (38). The concomitant presence of all these operational features in one single device is fundamental considering the overlapping medical conditions newborns may display right after birth (6).

Transport incubators are usually closed, small and minimalistic in their functionality, characterized by intuitive design and interfaces (38).

The incubator market is moderately competitive, as only a handful of major manufacturers, like GE Healthcare and Natus Medical Incorporated (US), Drägerwerk AG & Co. KGaA (Germany), Atom Medical Corporation (Japan), and Phoenix Medical Systems (Pvt) Ltd. (India) account for the most part of its share^4^. The cost of standard incubators varies considerably, spanning form 1,500 U$ (for some transport incubators) to 50,000 U$ (for some closed NICU incubators) (38, 39).

The state-of-the-art devices of GE Healthcare, such as the Giraffe OmniBed Carestation Incubator, the Giraffe and Panda warmer, and the Giraffe shuttle, are high-tech stationary or transport incubators which are characterized by complex design and high price. For these reasons, most “western” incubators are not suitable for LMICs and SSA. Increased complexity often comes with technical faults alongside the need for additional qualified training, which overall further increase their market price (38).

The price range for a simple transport incubator, without taking into account equipment for monitoring or respiratory support, such as the GE Healthcare Lullaby, the INC-TRP from SS Technomed, the TINC-101 from Phoenix Medical Systems, the BT-100 from Zhengzhou Dison Instrument and Meter Co. and the Isolette C2000 from Dräger is on a different scale (from 400 U$ to 10,000 U$) compared to stationary incubators^5^. The Dräger C2000 is one of the 25 best-selling incubators worldwide as it includes thermo-monitoring system and servocontrol (40). The thermo-monitoring allows the concomitant display of a central and peripheral temperature potentially allowing detection of cold stress before hypothermia (40).

### The Issue of Hypothermia in SSA

#### The Adherence to the WHO Warm Chain Procedures for Prevention

The WHO warm chain recommendations are available since almost 25 years, but neonatal hypothermia persists and still kills in SSA (1–4). Hence, these guidelines are either insufficient, inappropriate, or not followed. Some elements of the warm chain may indeed not be applicable in SSA settings. This is for instance partly the case for the environmental temperature at delivery, whether in hospitals or households, that can hardly be controlled to meet the recommendations, remaining strongly exposed and dependent on diurnal and seasonal variations (41). Moreover, the prompt drying and wrapping, placing the infant onto the mother's chest for skin-to-skin contact, and the early initiation of breastfeeding lack consistent practice (41). In rural regions of SSA, both healthcare providers and mothers-to-be have little adequate knowledge of neonatal physiology, appropriate handling, and even simple existing thermal care solutions (13, 41).

Hypothermia appears to represent a major challenge particularly for premature and low-birth-weight newborns, whose births are rising worldwide. About 15 million babies are being born prematurely each year and the highest incidence is in SSA and developing countries^6^.

In physiologic terms, the skin of preterm/LBW newborns is immature and has little superficial protection and underlying fat insulation, providing only a limited heat barrier; moreover, LBW infants have a higher ratio of body surface to weight, which physically increases heath loss. Finally, LBW and preterm newborns have only low brown adipose tissue (BAT) storage. BAT is built up during the last weeks of gestation as the first line of metabolic non-shivering thermogenesis in the term newborn (42). Altogether, LBW and premature newborns can produce less heat while being more susceptible to lose it, and therefore, are more prone to cold stress and hypothermia (42). Re-warming neonates highly relies on technology-based solutions.

#### The Operation of Essential Technologies to Treat Hypothermia in SSA

In SSA, hospitals are most often underfunded and devoid of continuous electricity to guarantee medical device operation (43). Their frequent power cuts place SSA countries among the 20 main electricity access-deficit countries. Moreover, access to electricity does not imply reliability neither good quality, and unscheduled interruptions and voltage fluctuations frequently occur (43). Adequate and reliable electric power supply is vital for standard medical devices and incubators since they are made of complex electronic circuits. Thus, by triggering technical malfunctioning, unstable power supply is in part responsible for newborn mortality by underheating or overheating.

As universally conceived in western countries, market-available infant incubators are ill-adapted for SSA and its unstable electricity profile (44, 45). In SSA, almost all medical devices are imported, either brand-new or regenerated. Despite initial compliance with operational requirements, equipment half-lives are very short because of power cuts, poor maintenance, shortage of spare parts, and improper use (44, 45). Accordingly, about 70% of LMICs' medical devices as partially or completely non-functional (45).

#### Infant Incubators and Radiant Warmers in Place in SSA

In SSA, the technology-based solutions to treat newborn hypothermia must be robust and affordable. Hence, low-cost devices have been developed in the 90s considering the limited availability of resources in SSA and, more in general, in LMICs.

The first low-cost incubator for these settings was developed in Uganda in 1968 by Oscar van Hemel. This closed incubator, named HEBI (HEmel Baby Incubator), was inexpensive, effective, and easy to assemble and repair. Over 1,400 HEBI incubators have been supplied and are still in use in LMICs^7^. However, the obsolete technology together with the dependence on the Dutch market for shipping and repair, gradually led to the termination of the HEBI production. In 2010, the Baltimore Kiwanis Incubator Foundation (BKIF) developed a similar closed incubator for Central and South American countries. BKIF incubators are low-cost and light, displaying a compact and high-tech design which employs long-lived incandescent light bulbs^8^. A low-cost thermal bag, called Embrace, has been conceived around 2010 and distributed to LMICs by Phoenix Medical Systems from 2016^9^. Embrace is proven to be cost-effective, small, portable, and safe. However, its pouch-like design does not include any system to monitor the temperature. Moreover, issues have been reported in terms of access to the baby, cleaning, and charging (46). The Hot Cot incubator, provided by Inditherm Medical CosyTherm™, is an example of a low-cost and simple model standardly employed in Malawian hospitals. Limitations are mainly related to the fact that this device is not equipped with automated servocontrol (47). In 2010, the Car Part incubator was conceived and essentially made of old car parts. This low-cost design relied on components belonging to obsolete vehicles, which in developing contexts turned out to be expensive and not easy to retrieve. At present, the design entered in disuse mostly for this reason, together with others related to the limited access to the baby and its complex assembly^10^. In 2014, James Roberts won the Dyson Award for a low-cost, portable, and inflatable infant incubator named mOm, that works with batteries and is highly portable. It is even equipped with a screen displaying temperature and humidity. The mOm incubator has a robust design and is currently in the process of being CE marked in Europe as Class IIb device^11^. Recently, a low-cost incubator made of a cardboard chamber has been designed at UMBC and is being field-tested (48). This incubator has been conceived to be halfway disposable after use (the cardboard chamber), and halfway reusable (the heating unit). Its design allows it to be easily transportable and straightforward to assemble, although issues related to robustness and cleaning have been reported (48)^12^. A more recent device is the AUI-Techno, an example of locally designed incubator conceived by the Cameroonian engineer Serge Armel Njidjou. It's local conception and design have been developed to cope with tropical weather and electric power outages, using solar energy as back-up power^13^.

### A Special Focus on Hypothermia's Prevention and Treatment in Kenya

In Kenya, the current population is about 55 million people, with a growth rate increase of 2.26% from 2020 to 2021^14^. Importantly, 75% of the population lives in rural areas, which undoubtfully provide less healthcare services for both quantity and quality. In Kenya, 21 newborn deaths per 1,000 live births have been reported in 2019 (4), way above the SDG3.2 target of ≤ 12 deaths every 1,000 live births (see text footnote 2). Considering this rate and that about 1,500,000 live births were officially recorded in the country register, at least 31,500 newborns must have died in 2019^15^.

About 50% of the Kenyan population lives in condition of poverty, which implies that children are born in poor households with little access to healthcare services and deliveries that occurs most frequently at home (49, 50). Moreover, in rural Kenya, 16 million people out of the 55 million total population lack access to electricity (43).

In a prospective study conducted in 2016, an overall insufficient adherence to the WHO warm chain protocol to prevent hypothermia was significantly associated with newborn mortality (12). This sub-optimal compliance is attributable to a fundamental mismatch between the needs of the population and the offer of services by the healthcare across the country, where a general shortage in human and financial resources is highly noted. In fact, a study conducted among Kenyan counties in 2020 highlighted the lack of physicians and nurses^16^.

Specifically, these studies showed that the temperature of the delivery rooms was consistently below the WHO recommendations, almost 50% of the mothers were not educated for newborn thermal care, and rooming in, skin-to-skin contact, and early breastfeeding were often ignored, with an adherence percentage of 10% only for all of them individually. Of note, however, 81.7% of newborns were promptly dried and wrapped, thermal resuscitation practices were observed (64%), as well as early bath avoidance (74%) (12).

In a survey performed in 2018 across 31 healthcare facilities within the county of Nairobi, a shortage of essential infant equipment has been clearly revealed, including warming and resuscitation devices to treat hypothermia (49). Furthermore, a subsequent inspection conducted at the Kenyatta National Hospital (KNH) brought to light that even higher-level Kenyan hospitals were *de facto* unable to provide adequate maintenance and repair services for technical medical equipment (51).

Several strategies have been proposed to promote local production and research to provide SSA with essential technologies. As an example, the Maker Movement for “Maternal, Newborn and Child Health” has been launched in 2013 to empower local partnerships at KNH and foster collaboration with the Kenya National Bureau of Standards for technical guidance, international regulatory frameworks, and staff training (51). Of note, the regulatory capacity for medical devices is very limited in SSA, with only South Africa disposing of regulatory frameworks that are internationally recognized (52). Importantly, this lack of available materials and protocols is limiting the local fabrication capacity, precluding SSA's independence from the international markets (52).

## Discussion and Conclusions

### The Framework of Current Knowledge

Neonatal mortality in SSA is a major burden and a tragic reality which can be summarized in the most part as “*too many tiny babies and not enough medical equipment to provide life-saving newborn care”* (45). Here, the combination of knowledge gaps as well as a general distrust for the current hypothermia prevention strategies make technology-based solutions for treatment particularly indispensable. One key example is represented by the skin-to-skin practice of KMC, an essential measure for the WHO warm chain, which is fundamental for both mother-baby interaction and newborn development. Importantly, KMC also contributes to a substantial reduction of neonatal mortality by hypothermia and is highly effective for LWB and premature newborns. Nevertheless, KMC is insufficiently accepted and practiced in SSA, because of several features, such as poverty, cultural beliefs, and the poorly developed neonatal care units.

Despite being the first key intervention to prevent hypothermia, KMC cannot last 24 h and is unfeasible, for instance, when the mother needs to rest; moreover, KMC becomes also impractical in case of unstable newborns that necessitate constant monitoring. For these reasons, technology-based solutions most be in place as a complementary tool to KMC “for” the mother and the infant and not “between” them.

Even though essential, the shortage of robust and affordable incubators to treat hypothermia and their frequent improper operation when available must be considered as a significant contributing factor for neonatal death (together with prematurity, asphyxia, respiratory distress syndrome, and infections).

Access to solid and performing technologies may significantly reduce the burden of neonatal disease and mortality (53). Infant medical devices are in fact core components of healthcare development and represent unavoidable building blocks for sustainable health progress. The demand for low-cost and high-tech infant medical equipment remains high, as many healthcare facilities call for efficient and adapted equipment to deliver the quality and quantity of healthcare services needed.

Local ministries of health need to invest more in the perinatal sector, improve the quality of maternal, antenatal, and postnatal care. By strengthening neonatal medicine and neonatal care in fact, they will also promote engagement and empowerment of mothers and mothers-to-be, couples, families, and communities.

All in all, there is an urgent need for holistic, yet affordable, technology-based interventions to sensitize health workers, produce knowledge, promote awareness, and ultimately save lives in countries which desperately need so. For SSA countries to be able to adopt a large-scale local production of medical devices, regulatory institutions and frameworks should be established to parallel the local fabrication capacity, ultimately allowing SSA's independence from the overseas market.

Evidence reinterpretation: call for research focused on technology-based solutions tailored to the SSA context, the need, and the end-user.

It is of paramount importance that medical devices for LMICs are specifically designed to cope with their harsh settings. Inputs form the local context, need, and end user are needed to design equipment that will enable the provision of skilled care to newborns. Given the unstable power profiles and the critical aspects related to large-scale production and maintenance, it is highly necessary to research for robust, energy efficient, low-cost, tropicalized, user-friendly solutions adapted to local protocols (Table 1). In addition, smart-tech equipment and incubators will allow standardized collection of data on thermal care, which is indispensable for the generation of clinical and epidemiological evidence as well as for raising medical awareness. Such data can allow the gathering of evidence-based information for policy-makers and boost prevention- and treatment-focused interventions in the next future.

**Table 1:** Essential features for incubators tailored to the SSA contexts, needs, and end-users.

**Features**	**Context**	**Research output**
Affordability	∘ Underfunded health care infrastructures	Low-cost
Robustness	∘ Inadequate maintenance and repair, ∘ Shortage of spare parts	Smart-tech (essential design, low complexity)
Focused on the end-user	∘ Inadequate training ∘ Staff shortage	Intuitive assembly, handling, and operation
Based on local needs	∘ Local medical protocols ∘ Newborn admissions	Fit to local protocols and infant admission loads
Energy efficiency	∘ Power outages ∘ Lack of access to electricity ∘ Inadequate quality of electricity	Design to cope with local electricity profiles; renewable energy
Compatible with the local manufacture	∘ Local production capacity ∘ Component availability ∘ Transport efficiency ∘ Sustainability	Empowerment of the local production
Easy cleaning	∘ Infections ∘ Infrastructure gaps (i.e., inadequate sterilization)	Safe and manageable
Resistant to the tropical environment	∘ Harsh environment (humidity, temperature, and dust)	Guaranteed operation in the local environment
Certified	∘ Lack of local institutions for regulatory framework (52)	CE/FDA approval overseas

## Summary

- Neonatal mortality in SSA is a major burden.
- The lack of robust and affordable incubators to treat hypothermia must be considered as determinant of neonatal death.
- Gaps in the knowledge and distrust around the measures to prevent hypothermia make technology-based solutions for treatment indispensable.
- Technology-based solutions must be in place as a tool “for” and not “between” the mother and the infant.
- Medical devices in SSA are imported and not specifically designed to cope with its harsh settings.
- Inputs form the local contexts, and end-users are necessary, to design equipment that will enable the provision of skilled care to newborns.
- Access to solid and performing technology-based solutions may significantly reduce the burden of neonatal disease and mortality in SSA.

## Data Availability Statement

The original contributions presented in the study are included in the article/supplementary material, further inquiries can be directed to the corresponding author.

## Author Contributions

GBP, CG, SS, MAR, SM, KS, MR-K, MK, and REP contributed to the conception and design of the work, alongside with the acquisition, analysis, and the data interpretation, drafting and editing of the work content, the final approval of the revised version for publication, and ensured accuracy and integrity. All authors contributed to the article and approved the submitted version.

## Funding

GBP, CG, SS, MAR, SM, and KS are supported by a grant from Tech4Dev, an internal funding program from EPFL.

## Conflict of Interest

The authors declare that the research was conducted in the absence of any commercial or financial relationships that could be construed as a potential conflict of interest.

## Publisher's Note

All claims expressed in this article are solely those of the authors and do not necessarily represent those of their affiliated organizations, or those of the publisher, the editors and the reviewers. Any product that may be evaluated in this article, or claim that may be made by its manufacturer, is not guaranteed or endorsed by the publisher.

## Footnotes

^1^ https://sdgs.un.org/goals

^2^ https://unstats.un.org/sdgs/report/2020/goal-03/

^3^All countries to the south of the Sahara desert: Angola, Benin, Botswana, Burkina Faso, Burundi, Cabo Verde, Cameroon, Central African Republic, Chad, Comoros, Congo, Côte d'Ivoire, Democratic Republic of the Congo, Djibouti, Equatorial Guinea, Eritrea, Eswatini, Ethiopia, Gabon, Gambia, Ghana, Guinea, Guinea-Bissau, Kenya, Lesotho, Liberia, Madagascar, Malawi, Mali, Mauritania, Mauritius, Mozambique, Namibia, Niger, Nigeria, Rwanda, Sao Tome and Principe, Senegal, Seychelles, Sierra Leone, Somalia, South Africa, South Sudan, Togo, Uganda, United Republic of Tanzania, Zambia and Zimbabwe.

^4^ https://www.mordorintelligence.com/industry-reports/incubators-devices-market

^5^ https://bimedis.com/search/search-items/neonatology-neonatal-incubators

^6^ https://www.who.int/news-room/fact-sheets/detail/preterm-birth

^7^ https://hebi-incubator.org/

^8^ http://bkif.org/incubator-specifications/

^9^ https://www.embraceglobal.org/

^10^ https://www.financialexpress.com/archive/a-baby-incubator-made-from-car-parts/716637/

^11^ https://www.momincubators.com/

^12^ https://news.umbc.edu/low-cost-infant-incubator-developed-at-umbc-completes-successful-clinical-trial-in-india/

^13^ https://www.afd.fr/en/actualites/made-cameroon-specially-designed-incubators-premature-babies

^14^ https://www.macrotrends.net/countries/KEN/kenya/population-growth-rate

^15^ https://knoema.com/atlas/Kenya/topics/Demographics/Fertility/Number-of-births

^16^Mohiddin A and Temmerman M, “COVID-19 Exposes Weaknesses in Kenya's Healthcare System and What Can Be Done” (The Conversation) < http://theconversation.com/covid-19-exposes-weaknesses-in-kenyas-healthcaresystem-and-what-can-be-done-143356> accessed 9 September 2021.

## References

[1] Jamison DT Murphy SM Sandbu ME. Why has under-5 mortality decreased at such different rates in different countries? J Health Econ. (2016) 48:16–25. 10.1016/j.jhealeco.2016.03.002 27046447 PMC4921600

[2] The Lancet Global Health. Progressing the investment case in maternal and child health. Lancet Glob Health. (2021) 9:e558. 10.1016/S2214-109X(21)00178-9 33865462

[3] World Health Organization. Human Resource Strategies to Improve Newborn Care in Health Facilities in Low- and Middle-Income Countries. Originally published under the title “Roadmap on Human Resource Strategies to Improve Newborn Care in Health Facilities in Low- and Middle-Income Countries”. © Geneva: World Health Organization (2020).

[4] United Nations Child Mortality. Levels & Trends in Estimates developed by the UN Inter-agency Group for Child Mortality Estimation (UN IGME). Washington, DC: United Nations Child Mortality (2020).

[5] World Health Organization. WHO and Maternal and Child Epidemiology Estimation Group (MCEE) estimates for child causes of death 2000–2017 produced in August 2019. Avilable online at: https://www.who.int/gho/child_health/mortality/causes/en/#

[6] Lunze K Bloom DE Jamison DT Hamer DH. The global burden of neonatal hypothermia: systematic review of a major challenge for newborn survival. BMC Med. (2013) 11:24. 10.1186/1741-7015-11-24 23369256 PMC3606398

[7] WHO. WHO_RHT_MSM_97.2_Thermal protection of the Newborn.pdf. Geneva: WHO (1997).

[8] Mullany LC Katz J Khatry SK LeClerq SC Darmstadt GL Tielsch JM. Risk of mortality associated with neonatal hypothermia in southern Nepal. Arch Pediatr Adolesc Med. (2010) 164:650–6. 10.1001/archpediatrics.2010.103 20603466

[9] Maternal health safe motherhood programme division of family health world health organization Geneva. Practical Guide. (1993). Available online at: https://apps.who.int/iris/bitstream/handle/10665/60042/WHO_FHE_MSM_93.2.pdf;se

[10] Coalter WS Patterson SL. Sociocultural factors affecting uptake of home-based neonatal thermal care practices in Africa: a qualitative review. J Child Health Care. (2017) 21:132–41. 10.1177/1367493516686201 29119805

[11] Kumar V Shearer J Kumar A. Neonatal hypothermia in low resource settings: a review. J Perinatol 29, 401–412. (2009). 19158799 10.1038/jp.2008.233

[12] Nyandiko WM Kiptoon P Lubuya FA. Neonatal hypothermia and adherence to World Health Organisation thermal care guidelines among newborns at Moi teaching and referral hospital, Kenya. PLoS ONE. (2021) 16:e0248838. 10.1371/journal.pone.0248838 33755686 PMC7987163

[13] Kyokan M Jirapaet V Rosa-Mangeret F Pisoni GB Pfister RE. Gaps in neonatal thermal care in low-resource settings: a web-based survey of healthcare workers. Research Square. (2021). 10.21203/rs.3.rs-980430/v1 PMC9511651 36645785

[14] Beletew B Mengesha A Wudu M Abate M. Prevalence of neonatal hypothermia and its associated factors in East Africa: a systematic review and meta-analysis. BMC Pediatr. (2020) 20:148. 10.1186/s12887-020-02024-w 32245438 PMC7118870

[15] Soll R. Heat loss prevention in neonates. J Perinatol. (2008) 28:S57–9. 10.1038/jp.2008.51 18446179

[16] McCall EM Alderdice F Halliday HL Vohra S Johnston L. Interventions to prevent hypothermia at birth in preterm and/or low birth weight infants. Cochrane Database Syst Rev. (2018) 2:CD004210. 10.1002/14651858.CD004210.pub5 29431872 PMC6491068

[17] DE Simkiss. Kangaroo mother care. J Trop Pediatr. (1999) 45:192–4. 10467828 10.1093/tropej/45.4.192

[18] Conde-Agudelo A Belizán JM Diaz-Rossello J. Kangaroo mother care to reduce morbidity and mortality in low birthweight infants. Cochrane Database Syst Rev. (2011) CD002771. 10.1002/14651858.CD002771.pub2 21412879

[19] Salim N Shabani J Peven K Rahman QS Kc A Shamba D. Kangaroo mother care: EN-BIRTH multi-country validation study. BMC Pregnancy Childbirth. (2021) 21:231. 10.1186/s12884-020-03423-8 33765950 PMC7995571

[20] Kinshella MW Hiwa T Pickerill K Vidler M Dube Q Goldfarb D. Barriers and facilitators of facility-based kangaroo mother care in sub-Saharan Africa: a systematic review. BMC Pregnancy Childbirth. (2021) 21:176. 10.1186/s12884-021-03646-3 33663415 PMC7934357

[21] World Health Organization. WHO Recommendations on Interventions to Improve Preterm Birth Outcomes. (2015). Available online at: https://www.who.int/reproductivehealth/publications/maternal_perinatal_health/preterm-birth-guideline/en/26447264

[22] WHO Immediate KMC Study Group Arya S Naburi H Kawaza K Newton S Anyabolu CH. Immediate “Kangaroo Mother Care” and survival of infants with low birth weight. N Engl J Med. (2021) 384:2028–38. 10.1056/NEJMoa2026486 34038632 PMC8108485

[23] Baillet A,. Jugemens des Savans sur les Principaux Ouvrages des Auteurs. French Translators, 1600–1800: An Online Anthology of Prefaces Criticism. (1722) 6. Retrieved from: https://scholarworks.umass.edu/french_translators/6

[24] Hess JH. Premature and Congenitally Diseased Infants. Philadelphia PA: Lea & Febiger (1922). p. 205–33.

[25] Cone TE. The first published report of an incubator for use in the care of the premature infant (1857). Arch Pediatr Adolesc Med. (1981) 135:658. 7018217 10.1001/archpedi.1981.02130310062020

[26] Rebovich Kelsey. The Infant Incubator in Europe (1860-1890). (2017). Embryo Project Encyclopedia. Available online at: http://embryo.asu.edu/handle/10776/11407/

[27] Budin PC. Le Nourisson. Paris: Octave Doin (1900). (English translation by Maloney WJ. The Nursling. London: Caxton Publishing (1907).

[28] Stern L. The newborn infant and his thermal environment. Curr Probl Pediatr. (1970) 1:1–29. 4950535

[29] LeBlanc MH. Thermoregulation: incubators, radiant warmers, artificial skins, and body hoods. Clin Perinatol. (1991) 18:403–22. 1934849

[30] Silverman WA Blanc WA. Effect of humidity on survival of newly born premature infants. Pediatrics. (1957) 20:447. 13465237

[31] Agate FJ Silverman WA. The control of body temperature in the small newborn infant by low-energy infra-red radiation. Pediatrics. (1963) 31:725–33. 14011200

[32] Silverman WA. Human Experimentation: A Guided Step Into the Unknown. New York, NY: Oxford Medical Publishers (1985). p. 178.

[33] Perlstein PH Edwards NK Sutherland JM. Apnea in premature infants and incubator air temperature changes. N Engl J Med. (1970) 282:461–6. 5411868 10.1056/NEJM197002262820901

[34] Atherton HD Edwards NK Perlstein PH. A computerized incubator control system for newborn infants. In: Computer Technology to Reach the People. New York, NY: IEEE (1975).

[35] Perlstein PH Edwards NK Atherton HD Sutherland JM. Computer assisted newborn intensive care. Pediatrics. (1976) 57:494–501. 1264544

[36] Philip A. The evolution of neonatology. Pediatr Res. (2005) 58:799–815. 15718376 10.1203/01.PDR.0000151693.46655.66

[37] Flenady V Woodgate PG. Radiant warmers versus incubators for regulating body temperature in newborn infants. Cochrane Database Syst Rev. (2003). CD000435. 10.1002/14651858.CD000435 14583922

[38] Braun G., Hentschel R. Incubators. In: Kramme R, Hoffmann KP, Pozos RS, editos. Springer Handbook of Medical Technology. Berlin: Springer (2011).

[39] Lunze K Hamer D. Thermal protection of the newborn in resource-limited environments. J Perinatol. (2012) 32:317–24. 22382859 10.1038/jp.2012.11

[40] Andrew Lyon Peter Püschner. ThermoMonitoring: A Step Forward in Neonatal Intensive Care. Available online at: https://www.draeger.com/Library/Content/thermomonitoring-bk-9097384-us.pdf

[41] Onalo R. Neonatal hypothermia in sub-Saharan Africa: a review. Niger J Clin Pract. (2013) 16:129–38. 10.4103/1119-3077.110120 23563449

[42] Symonds ME Bloor I Ojha S Budge H. The placenta, maternal diet and adipose tissue development in the newborn. Ann Nutr Metab. (2017) 70:232–5. 10.1159/000464301 28301844

[43] IEA IRENA UNSD World Bank WHO. Tracking SDG 7: The Energy Progress Report. (2021). Washington, DC: World Bank. © World Bank. License: Creative Commons Attribution—NonCommercial 3.0 IGO (CC BYNC 3.0 IGO).

[44] World Health Organization. Medical Devices: Managing the Mismatch. An Outcome of the Priority Medical Devices Project. Geneva: World Health Organization (2010).

[45] Richards-Kortum RR. Tools to reduce newborn deaths in Africa. Health Aff . (2017) 36:2019–22. 10.1377/hlthaff.2017.1141 29137526

[46] Nimbalkar S Patel H Dongara A Patel DV Bansal S. Usage of EMBRACE™ in Gujarat, India: survey of paediatricians. Adv Prev Med. (2014) 2014:415301. 10.1155/2014/415301 25530887 PMC4230002

[47] Zwiener P Zhang M Smith M Romeo R Charnsangavej L. Hot Cot Team Binder (2009).

[48] Arnold, C, NEWS, FEATURE, 12 November, 2019, Correction 29 November, 2019,. Who Shrank the Drug Factory? Briefcase-Sized Labs Could Transform Medicine. Available online at: https://www.nature.com/articles/d41586-019-03455-x 10.1038/d41586-019-03455-x 31719703

[49] Murphy GAV Gathara D Abuya N Mwachiro J Ochola S Ayisi R. What capacity exists to provide essential inpatient care to small and sick newborns in a high mortality urban setting? - A cross-sectional study in Nairobi city county, Kenya. PLoS ONE. (2018) 13:e0196585. 10.1371/journal.pone.0196585 29702700 PMC5922525

[50] Republic Republic of Kenya Ministry Ministry of Health. Newborn Care Pocket Book for Primary Healthcare Workers in Dispensaries and Health Centres. Nairobi: HECTA Consulting Ltd. (2018).

[51] Ayah R Ong'ech J Mbugua EM Kosgei RC Waller K Gathara D. Responding to maternal, neonatal and child health equipment needs in Kenya: a model for an innovation ecosystem leveraging on collaborations and partnerships. BMJ Innov. (2020) 6:85–91. 10.1136/bmjinnov-2019-000391 32685187 PMC7361008

[52] NEPAD. Medical Device Regulations in Southern Africa Will Boost Innovation and Improve Patient Health. Johannesburg: NEPAD (2019).

[53] Makohliso S Klaiber B Sahli R Tapouh JRM Amvene SN Stoll B. Medical Technology Innovation for a Sustainable Impact in Low- and Middle-Income Countries: A Holistic Approach. (2020). 10.31224/osf.io/2dytg

